# Regulation of Inflammation in Autoimmune Disease

**DOI:** 10.1155/2019/7403796

**Published:** 2019-02-28

**Authors:** Lihua Duan, Xiaoquan Rao, Keshav Raj Sigdel

**Affiliations:** ^1^Department of Rheumatology and Clinical Immunology, Jiangxi Provincial People's Hospital, Nanchang 330006, China; ^2^School of Medicine, Case Western Reserve University, Cleveland 44106, USA; ^3^Department of Internal Medicine, Patan Academy of Health Sciences, Patan 44700, Nepal

Inflammation is a normal physiological defense against pathogen infection and tissue damage and quickly ends under normal circumstances. However, in many chronic conditions, the inflammatory response continues and leads to significant tissue and organ damage. Recently, increasing evidences have shown that the abnormal inflammatory response is closely associated with many chronic diseases, especially in autoimmune diseases, including rheumatoid arthritis (RA), inflammatory bowel disease (IBD), systemic lupus erythematosus (SLE), gout, and diabetes [1–3]. Although the importance of inflammatory dysregulation in chronic illnesses has been reported in recent studies, the pathogenesis of inflammation dysfunction in the autoimmune diseases remains elusive. Knowledge of the mechanism of inflammation regulation will lead to significant clinical benefits for the treatment of autoimmune disease. This special issue showcases a number of original research articles and review papers on the topic of inflammatory regulation in autoimmune diseases.

T cell-mediated inflammatory responses have long been recognized to play an essential role in the development of autoimmune diseases, including Th1, Th2, and Th17 cell responses. Recent compelling evidence has shown that abnormal T cell immune response, including Th1, Th2, and Th17 cell responses, was actually having a crucial role in the inflammation of autoimmune diseases [4]. Recent studies showed that vasoactive intestinal peptide (VIP) modulates the pathogenic activity of diverse cell subpopulations involved in RA, including lymphocytes, fibroblast-like synoviocytes (FLS), and macrophages [5]. In this special issue, R. Villanueva-Romero et al. summarized the anti-inflammatory and immunomodulatory actions of VIP on T cell function in RA. The costimulatory molecule dyad interaction between T cells and APCs has been linked to the development of abnormal immune response [6]. Therefore, inhibition of costimulatory molecule interaction has been suggested to result in impaired T cell activation. Here, R. O'Dwyer et al. introduced a specific anti-ICOSL new antigen receptor domain which significantly alleviated the inflammation of joints and delayed and reduced overall disease progression and severity in a mouse model of RA by blocking the ICOS/ICOSL interaction and inhibiting T cell proliferation. S. Lilliebladh et al. found that the CCL20 concentrations and percentages of Th17 cells were increased in anti-neutrophil cytoplasmic antibody- (ANCA-) associated vasculitide (AAV) patients. Consistently, Y. Sun et al. also reported that the level of IL-17, the important cytokine of Th17, was also elevated in Sjögren's syndrome patients. Numerous studies revealed that Tregs exert a critical role in immune tolerance and play a protective role in the autoimmune diseases [7]. Here, J. Sun et al. stably induced human CD8+ regulatory T cells (hCD8+ Tregs) by TGF-*β*1 and rapamycin (RAPA). Importantly, these hCD8+ Tregs could significantly alleviate the severity of collagen-induced arthritis. In addition to TGF-*β*1 and RAPA, J. Zhu et al. reported that cytokine IL-33 can also protect mice against DSS-induced chronic colitis by increasing regulatory T cell responses and inhibiting Th17 cell response. S. Zhang et al. meta-analyzed the proportions of Tregs present during the development of SLE. Besides, M. Morelli reported that the JAK inhibitor tofacitinib impaired the inflammatory effects of IFN-*γ* and IL-22 produced by Th1 and Th22 in the pathogenesis of psoriasis.

In addition to T cell, many other immune cells were also implicated in the development of autoimmune diseases. B cells are best known for their capacity to produce antibodies, which often play a deleterious role in the development of autoimmune diseases. B cell depletion is expected to alter B cell-mediated antibody production and cytokine secretion [8]. B. Yamout et al. demonstrated that rituximab was well tolerated and effective in reducing the relapse rate and disability progression in relapsing-remitting and progressive MS patients. Regulatory B cells (Bregs) were increasingly gaining attention for restraining inflammation through suppressing the differentiation of Th1 and Th17 immune responses in the development of autoimmune diseases [9, 10]. J. Zhu et al. here also demonstrated that IL-33 expanded Bregs in the DSS-induced colitis animal model. The innate immune cells monocytes/macrophages were considered as the important regulator in hepatic inflammation. H. Li et al. demonstrated that M1 macrophages promoted hepatic progenitor cell (HPC) self-renewing phenotype which was closely associated with Notch signaling activation. Toll-like receptor (TLR), a critical molecule of the innate immune system, plays a critical role in the development of autoimmune diseases. R. Shamilov and B. Aneskievich reviewed the regulation of TNIP1 on TLR signaling in autoimmune diseases. J. Sun et al. demonstrated that human amnion mesenchymal cells (hAMC) could attenuate the inflammation and promote the remyelination in EAE mice, which might be a promising cell source for the therapy of MS.

Epigenetic mechanism has been implicated in the development and progression of many autoimmune diseases [11]. In this special issue, X. Wang et al. showed that methylation variabilities among the same cytokines can greatly impact the perpetuation of the inflammatory process or signal pathway of autoimmune diseases, and differentiating the cytokine methylation status will contribute to understanding of the mechanisms of the diseases. Y. Li et al. reported that the activity of HDAC3 was reduced in peripheral blood mononuclear cells of patients with RA, while the acetylation of histone H3 was increased. J. G. Fernandes et al. analyzed the miRNA and gene expression profiles in peritoneal cells of AIRmax and AIRmin lines, and some miRNAs were significantly highly expressed in the pristine-induced arthritis-susceptible animals. Furthermore, many immune-mediated diseases showed gender and age difference. Y. Cao et al. reported that different genes in the Ifi200 family play different roles in sex difference in autoimmune diseases, and Y. Huang summarizes the relationship between inflammatory aging and premature ovarian insufficiency.

Collectively, all research and review articles in this special issue covers many important aspects in the area of inflammatory regulation in autoimmune diseases, which would provide some new ideas for diagnosis and treatment in these diseases.
